# Tenacibaculum platacis sp. nov., Tenacibaculum vairaonense sp. nov. and Tenacibaculum polynesiense sp. nov. isolated from batfish (Platax orbicularis) in Tahiti Island, French Polynesia

**DOI:** 10.1099/ijsem.0.006605

**Published:** 2025-01-06

**Authors:** Pierre Lopez, Benjamin Fradet, Lucie Coffion, Jean-François Bernardet, Denis Saulnier, Eric Duchaud

**Affiliations:** 1 Ifremer, IRD, Institut Louis‐Malardé, Université Polynésie française, EIO, F‐98719 Taravao, Tahiti, Polynésie Française, France; 2 Université Paris-Saclay, INRAE, UVSQ, VIM, Jouy-en-Josas, France; 3 LABGeM, Génomique Métabolique, CEA, Genoscope, Institut François Jacob, Université d’Évry, Université Paris-Saclay, CNRS, Evry, France

**Keywords:** aquaculture, *Flavobacteriaceae*, marine bacteria, *Platax orbicularis*, *Tenacibaculum*

## Abstract

Ten novel Gram-negative, aerobic, non-sporulating, yellow-pigmented rod-shaped bacterial strains motile by gliding were isolated from marine organisms/environments in French Polynesia. Three of them designated as 190524A05c^T^, 190524A02b^T^ and 190130A14a^T^ were retrieved from orbicular batfish (*Platax orbicularis*) mucus. Online database comparisons using 16S rRNA amplicons resulted in over 95% similarity to the genus *Tenacibaculum*. Phylogenetic analyses based on 679 concatenated core protein sequences revealed that strains 190524A05c^T^, 190524A02b^T^ and 190130A14a^T^ showed the highest similarity to *Tenacibaculum skagerrakense* DSM 14836^T^, *Tenacibaculum xiamenense* LMG 27422^T^ and *Tenacibaculum holothuriorum* S2-2^T^, respectively. Digital DNA–DNA hybridization and average nt identity values between strains 190524A05c^T^, 190524A02b^T^ and 190130A14a^T^ and other type strains were less than 76.25 and 24.1%, respectively. The DNA G+C content was 31.48, 30.66 and 31.98 mol% for strains 190524A05c^T^, 190524A02b^T^ and 190130A14a^T^, respectively. Menaquinone-6 was detected as the major isoprenoid quinone in these three strains. The major polar lipids (phosphatidylethanolamine and aminophospholipid) were similar to the chemotaxonomic profile of other species of the genus *Tenacibaculum*. Strain 190524A05c^T^ contained summed feature 3 (comprising C_16:1_ ω7c and/or iso-C_15:0_ 2-OH), iso-C_15:1_ G, iso-C_15:0_ and iso-C_17:0_ 3-OH as the major cellular fatty acids. Strain 190524A02b^T^ contained summed feature 3 (comprising C_16:1_ ω7c and/or iso-C_15:0_ 2-OH), iso-C_15:0_, iso-C_15:1_ G and iso-C_17:0_ 3-OH as the major cellular fatty acids. Strain 190130A14a^T^ contained iso-C_15:1_ G, summed feature 3 (comprising C_16:1_ ω7c and/or iso-C_15:0_ 2-OH), iso-C_15:0_ and iso-C_17:0_ 3-OH as the major cellular fatty acids. Based on the phenotypic and molecular features, these three strains represent novel species of the genus *Tenacibaculum* for which the names *Tenacibaculum platacis* sp. nov., with 190524A05c^T^ (= CIP 112470^T^ = DSM 118113^T^) as the type strain; *Tenacibaculum vairaonense* sp. nov., with 190524A02b^T^ (= CIP 112469^T^ = DSM 118112^T^) as the type strain; and *Tenacibaculum polynesiense* sp. nov., with 190130A14a^T^ (= CIP 112468^T^ = DSM 118111^T^) as the type strain, are proposed.

## Introduction

The genus *Tenacibaculum* (family *Flavobacteriaceae,* phylum *Bacteroidota*) was created by Suzuki *et al*. in 2001 through the reclassification of two bacterial species previously assigned to the genus *Flexibacter* [1]. At the time of writing, the genus comprises 35 validly named species (http://www.bacterio.net/tenacibaculum.html) [2], all retrieved from marine environments [3]. While most *Tenacibaculum* species are considered non-pathogenic, the type species *Tenacibaculum maritimum* (formerly *Flexibacter maritimus*) is a major fish pathogen causing considerable losses in marine fish farms worldwide. In addition, some other *Tenacibaculum* species recovered from the diseased or apparently healthy wild, feral and farmed fish have been shown or suspected to be pathogenic (i.e. *Tenacibaculum dicentrarchi*, *Tenacibaculum discolor*, *Tenacibaculum finnmarkense*, *Tenacibaculum gallaicum*, *Tenacibaculum ovolyticum*, *Tenacibaculum piscium* and *Tenacibaculum soleae*), collectively responsible for a condition known as tenacibaculosis [4].

In French Polynesia, tenacibaculosis affects farmed orbicular batfish (*Platax orbicularis*), a marine fish species naturally found in Polynesian lagoons [5]. At least one species, *T. maritimum*, is responsible for infectious episodes, but *Tenacibaculum mesophilum* can also be found on fish teguments. In order to better evaluate the diversity of *Tenacibaculum* species present in French Polynesian fish farms, a large sampling campaign was conducted between 2019 and 2021, focusing on bacterial isolates present at different times of the orbicular batfish production cycle and in different compartments such as water, farm structures or fish teguments [6]. This campaign resulted in the collection of more than 700 isolates, which were pre-identified by sequencing of the 16S rRNA V3–V5 region. Among them, a group of isolates formed a phylogenetic lineage within the genus *Tenacibaculum*. Here, we present a detailed taxonomic investigation of strains 190524A05c^T^, 190524A02b^T^ and 190130A14a^T^ using a polyphasic approach including genomic comparisons.

## Isolation of bacteria and habitat

During January 2019–June 2021, diseased and healthy orbicular batfish, water from different rearing locations, biofouling deposited on farm structures (net and jetfloat) and a variety of wild fish captured in the lagoon were sampled in French Polynesia. For bacterial isolation, gentle scrapping of fish body side or structures with sterile swab cotton-tipped applicators (COPAN) was performed. The water from the breeding tanks (1 to 2 l) was prefiltered using an 80-µm sieve. The filtrate was then passed over a membrane (0.2-µm porosity) using a vacuum pump. The membrane, placed in an Eppendorf tube containing 500 µl of sterile seawater, was grounded for 5 min in a bead breaker (30 Hz). Swabs and water filters were inoculated onto plates of *Flavobacteriaceae*-selective marine agar (FSMA) developed by an accredited veterinary diagnostic laboratory (Labofarm, Loudéac, France) as previously reported [5]. After 24- to 48-h incubation at 27 °C, colonies on FSMA were examined by phase-contrast microscopy, and those consisting of filamentous rods were sub-cultured and cryo-conserved at −80 °C in marine broth (MB) medium (M2216, Difco) supplemented with 15% glycerol. Bacterial strains included in this study are given in Table 1. In addition, strains *Tenacibaculum holothuriorum* DSM 113369^T^, *Tenacibaculum skagerrakense* DSM 14836^T^ and *Tenacibaculum xiamenense* LMG 27422^T^ were used as references for phenotypic characterization.

**Table 1.** *Tenacibaculum* strains used in this study

**Species**	Strains	Isolation source	Date of isolation	Site of isolation	GPS location	16S EzBioCloud (% id)	16S NCBI accession no.
*Tenacibaculum platacis*	190524A05c^T^	Tegument of an apparently healthy *Platax orbicularis*	24 May 2019	VAIA hatchery, Vairao, Tahiti Island	17° 48′ 23.4″ S 149° 17′ 32.6″ W	*T. skagerrakense* D30^T^ (98.82)	PP620846
	190611E02c	Tegument of an apparently healthy *Platax orbicularis*	11 June 21019	CRIOBE, Moorea Island	17° 31′ 08.4″ S 149° 51′ 00.5″ W	*T. skagerrakense* D30^T^ (98.82)	PP620849
	190820D02b	Biofooling from a fish net structure	20 August 2019	Net cage, Tautira, Tahiti Island	17° 49′ 51.9″ S 149° 07′ 43.6″ W	*T. skagerrakense* D30^T^ (98.82)	PP620850
	190607 A01a	Tegument of an apparently healthy *Platax orbicularis*	07 June 2019	Sea cage, Vairao, Tahiti Island	17° 48′ 23.1″ S 149° 17′ 35.9″ W	*T. skagerrakense* D30^T^ (98.82)	PP620851
*Tenacibaculum vairaonense*	190524A02b^T^	Tegument of an apparently healthy *Platax orbicularis*	24 May 2019	VAIA hatchery, Vairao, Tahiti Island	17° 48′ 23.4″ S 149° 17′ 32.6″ W	*T. aestuarii* SMK-4^T^ (97.43)	PP620847
	190607 A02c	Tegument of an apparently healthy *Platax orbicularis*	07 June 2019	Sea cage, Vairao, Tahiti Island	17° 48′ 23.1″ S 149° 17′ 35.9″ W	*T. aestuarii* SMK-4^T^ (97.50)	PP620852
	190115 A13a	Mucus of a fish belonging to the family *Acanthuridae*	15 January 2019	Sea cage, Vairao, Tahiti Island	17° 48′ 23.1″ S 149° 17′ 35.9″ W	*T. aestuarii* SMK-4^T^ (97.50)	PP620853
*Tenacibaculum polynesiense*	190130A14a^T^	Tegument of an apparently healthy *Platax orbicularis*	30 January 2019	VAIA hatchery, Vairao, Tahiti Island	17° 48′ 23.4″ S 149° 17′ 32.6″ W	*T. holothuriorum* S2-2^T^ (97.43)	PP620848
	190130 A13a	Tegument of an apparently healthy *Platax orbicularis*	30 January 2019	VAIA hatchery, Vairao, Tahiti Island	17° 48′ 23.4″ S 149° 17′ 32.6″ W	*T. holothuriorum* S2-2^T^ (97.43)	PP620854
	190423 A01a	Water from fish sea cage	23 April 2019	Net cage, VAIA hatchery, Vairao, Tahiti Island	17° 48′ 23.4″ S 149° 17′ 32.6″ W	*T. holothuriorum* S2-2^T^ (97.43)	PP620855

## 16S rRNA gene phylogeny

**Fig. 1.**
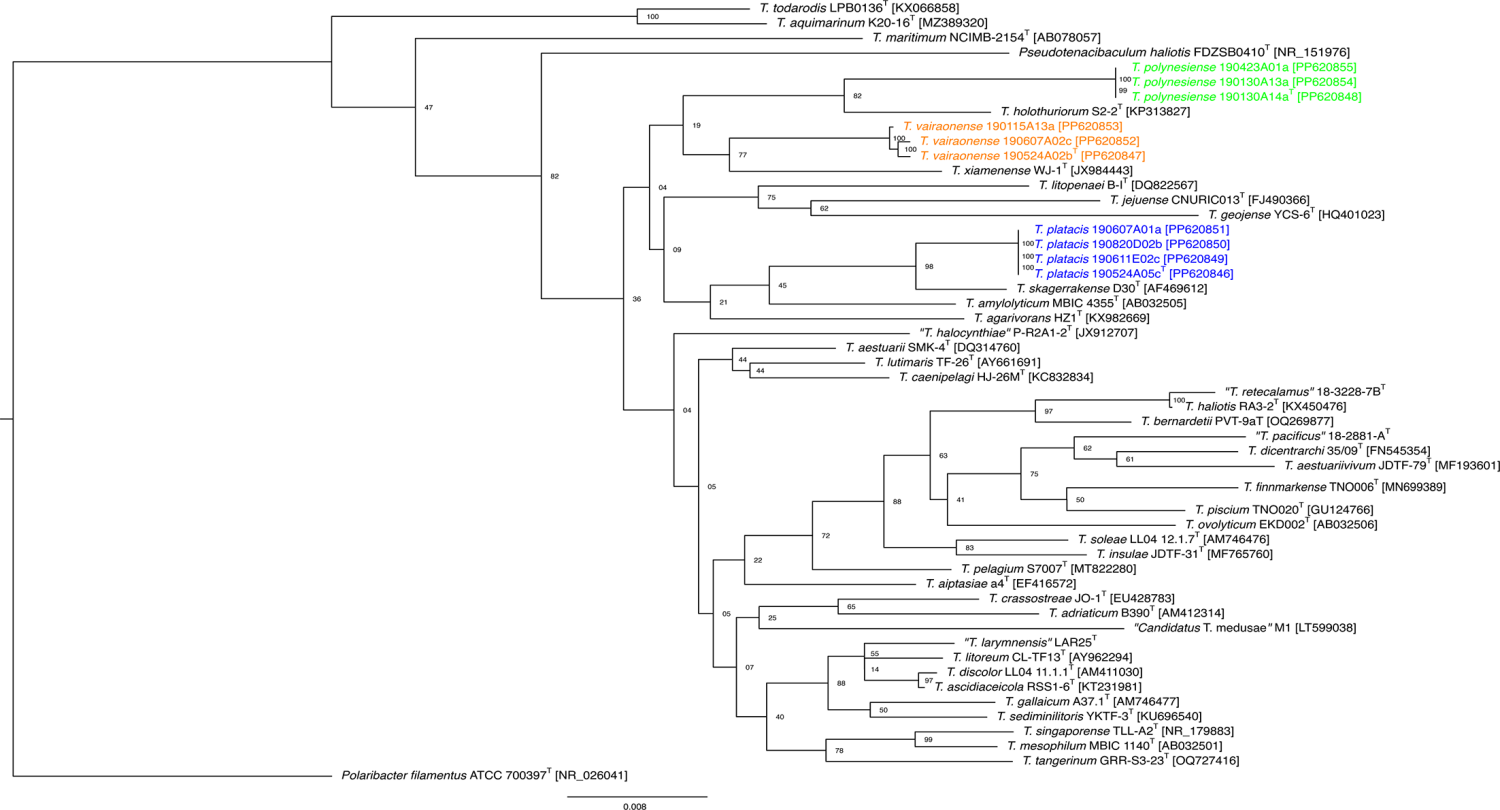
Neighbour joining 16S rRNA-based phylogeny. Phylogenetic tree inferred by FastME using the 16S rRNA gene sequences of *Tenacibaculum* species. The type strain of *Pseudotenacibaculum haliotis* has been included, and the type strain of *Polaribacter filamentus* was used as an outgroup. The scale bar indicates estimated sequence divergence, and bootstrap values are indicated at branch nodes. The 16S rRNA sequences of strains ‘*T. larymnensis*’ LAR25^T^, ‘*T. pacificus*’ 18–2881-A^T^ and ‘*T. retecalamus*’ 18-3228-7B^T^ have been retrieved from complete genome accession numbers GCF_028829235.1, NZ_CP115917 and NZ_CP115916, respectively.

Bacterial strains were grown in MB for 24 h at 27 °C and 170 r.p.m. Following centrifugation of the liquid culture, genomic DNA (gDNA) was extracted from the pellet. For PCR and Illumina sequencing, the Wizard gDNA purification kit (Promega, Madison, WI, USA) was used. For Nanopore sequencing, gDNA was extracted using a gDNA-Tip 100 G^−1^ system and buffer set (Qiagen). A rapid tentative taxonomic assignment was performed using nearly complete 16S rRNA gene sequences obtained by PCR and Sanger sequencing with the universal primers 27F (AGAGTTTGATCMTGGCTCAG) and 1492R (TACGGYTACCTTGTTACGACTT) and the EzBioCloud online 16S-based identification database [7]. The results (Table 1) suggest that ten selected strains likely belong to the genus *Tenacibaculum* with nt identity values between 97.50 and 98.82% with type strains of the genus. A tentative 16S rRNA-based phylogenetic tree was inferred by neighbour joining (Fig. 1) by the NGPhylogeny.fr suite [8] using MAFFT 7.407_1 alignment, Gblocks 0.91.1 curation and FastME 2.1.6.1_1 for tree inference with 1000 bootstrap replicates. A maximum likelihood-based tree was also constructed with the same data set but using PhyML+SMS 1.8.1_1 for tree inference (Fig. S1, available in the online version of this article). Both tree topologies confirmed the affiliation of these strains to the genus *Tenacibaculum*. Strains 190524A02b^T^, 190607 A02c and 190115 A13a clustered together with *T. xiamenense* WJ-1T^T^ as the closest related species. Strains 190130A14a^T^, 190130 A13a and 190423 A01a clustered together with *T. holothuriorum* S2-2^T^ as the closest related species. Strains 190524A05c^T^, 190611E02c, 190820D02b and 190607 A01a clustered together with *T. skagerrakense* D30^T^ as the closest related species. As previously reported [9,10], some nodes are poorly supported and *Pseudotenacibaculum haliotis* [11] is intertwined within the *Tenacibaculum* species. Indeed, the resolutive power of 16S rRNA-based phylogenies seems inefficient for delineating *Tenacibaculum, Pseudotenacibaculum* and *Polaribacter* species, some of which may need revision or reclassification [12].

## Genome features

Complete genomes were obtained for strains 190524A05c^T^, 190524A02b^T^ and 190130A14a^T^ using the hybrid assembly of Nanopore and Illumina reads, whereas draft genomes were obtained for the remaining strains using Illumina reads only. For short-read Illumina sequencing, the library was constructed using a TruSeq genomic kit and paired-end sequenced on a NextSeq instrument using a NextSeq 500/550 mid-output kit v2 (150 cycles). For long-read sequencing, gDNA was sequenced on GridION (Oxford Nanopore) using a flo-min106 (R9.4.1) flow cell. Assemblies were performed using Genome Assembly Service with default parameters (https://www.bv-brc.org/app/Assembly2) [13]. Genome characteristics are reported in Table S1. The resulting chromosomes or contigs (>2000 bp) were integrated into the MicroScope platform used for genome annotation, comparisons and core-genome computation [14]. Pairwise average nucleotide identity (ANI) values were computed using OrthoANI (https://www.ezbiocloud.net/tools/orthoani) [15], whereas digital DNA–DNA hybridization (dDDH) values were evaluated using Genome-to-Genome Distance Calculator v3.0 (GGDC; http://ggdc.dsmz.de/ggdc.php#) and Formula 2 [16] and are reported in Tables S2 and S3, respectively. ANI and dDDH values between strains 190524A05c^T^, 190524A02b^T^ and 190130A14a^T^ and other type strains were less than 76.25 and 24.1%, respectively. On the other hand, 190524A05c^T^, 190611E02c, 190820D02b and 190607 A01a strains displayed ANI values >96.44% and dDDH values >68.5%; 190524A02b^T^, 190607 A02c and 190115 A13a strains displayed ANI values >98.73% and dDDH values >88.2%; and 190130A14a^T^, 190130 A13a and 190423 A01a strains displayed ANI values >99.95% and dDDH values >99.5%.

**Table 2.** Differential phenotypic properties of *Tenacibaculum platacis* sp. nov. 190524A05c^T^, *Tenacibaculum vairaonense* sp. nov. 190524A02b^T^, *Tenacibaculum polynesiense* sp. nov. 190130A14a^T^, *T. skagerrakense* DSM 14836^T^, *T. xiamenense* WJ-1^T^ and *T. holothuriorum* DSM 113369^T^ The data presented in this table were determined in this study except for strain WJ-1^T^ (*T. xiamenense*), which are taken from Li et al., 2013 and strain ATCC 43398^T^ (*T. maritimum*), which are taken from Suzuki *et al.* 2001 [1]. +, Positive; −, negative; w, weak; nd, not determined.

Characteristic	*T. platacis* sp. nov. 190524A05c^T^	*T. vairaonense* sp. nov. 190524A02b^T^	*T. polynesiense* sp. nov. 190130A14a^T^	*T. skagerrakense* DSM 14836**^T^**	*T. xiamenense* WJ-1^T^	*T. holothuriorum* DSM 113369**^T^**	*T. maritimum* ATCC 43398**^T^**
**Colony appearance**							
Colony morphology	Circular, regular edge	Irregular, spreading edges	Circular, regular edges	Smooth, circular	Circular, regular edges	Smooth, circular	Flat, thin, uneven edge
Colony colour	Glistening, yellow-orange	Dark yellow-orange	Glistening, yellow	Yellow-orange	Bright yellow	Yellow	Pale yellow
Cell size	2 to 6 µm – 0.3 µm	5 to 10 µm – 0.5 µm	3 to 6 µm – 0.5 µm	2 to 8 µm – 0.3 µm	2 to 9 µm – 0.2 to 0.4 µm	4 to 8 µm – 0.5 µm	2 to 30 µm – 0.5 µm
**Grow range**							
pH	6.5–8.0	6.5–8.0	6.5–8.0	6.5–8.0	7.0–8.0	6.5–8.4	5.9–8.6
Temperature (°C)	18–34	18–34	18–34	15–37	16–38	18–34	15–34
NaCl (%)	1.5–5	1–4.5	1–4.5	0.5–5	2–4	1.5–5	1–3
**Metabolic test**							
Catalase	−	−	−	−	+	−	+
Nitrate reduction	−	−	−	−	+	−	+
**Hydrolysis of**							
Alginate	+	−	−	−	−	−	nd
Starch	+	−	+	+	+	+	−
Tween 20	+	+	w	+	nd	−	nd
Tween 60	+	+	+	+	nd	+	nd
Tween 80	+	+	+	+	+	+	+
Elastine	−	w	+	+	nd	−	nd
**Enzymatic activities(API ZYM**)							
Esterase C4	+	+	w	+	−	−	nd
*α*-Glucosidase	−	−	−	−	w	−	−
*β*-Glucosidase	−	−	−	−	+	−	−
*N*-Acetyl-*β*-glucosaminidase	−	−	−	w	+	−	−

**Table 3.** Fatty acid composition of *Tenacibaculum* strains 190524A05c^T^, 190524A02b^T^ and 190130A14a^T^ and the type strains of the closely related species of the genus Values are percentages of the total fatty acids, and fatty acids amounting less than 0.5% in all strains are not shown. The data presented in this table were determined in this study except for strain WJ-1T (*T. xiamenense*), which are taken from Li *et al.*, 2013 [23]. Major fatty acids (>5%) are shown in bold. nd, Not detected; tr, trace amount (<1.0%). The presence of unidentified fatty acids has not been represented in this table because they represent only traces compared to the other identified fatty acids.

Strain	*Tenacibaculum platacis* sp. nov. 190524A05c^T^	*Tenacibaculum vairaonense* sp. nov. 190524A02b^T^	*Tenacibaculum polynesiense* sp. nov. 190130A14a^T^	*T. skagerrakense* DSM 14836**^T^**	*T. xiamenense* WJ-1^T^	*T. holothuriorum* DSM 113369**^T^**
**Saturated fatty acids (SFAs**)						
C_14:0_	tr	tr	tr	tr	tr	tr
C_16:0_	tr	2.5	2.9	tr	2.5	tr
**Branched fatty acids**						
13:0 iso	tr	tr	1.1	tr	tr	tr
13:1 iso	nd	nd	tr	nd	tr	nd
14:0 iso	nd	tr	tr	tr	tr	tr
15:0 iso	**19.7**	**19.6**	**14.7**	**9.4**	**11.3**	**19.7**
15:0 anteiso	nd	1.5	1.1	tr	tr	tr
15:1 anteiso A=15:1 anteiso ω10c	nd	tr	tr	tr	nd	nd
15:1 iso G=15:1 iso ω10c	**20.7**	**15.7**	**21.5**	**11.8**	**4.9**	**9.0**
16:1 iso H (identified as 16:1 iso ω6c)	nd	nd	nd	nd	nd	3.1
16:0 iso	tr	1.7	2.1	1.1	tr	2.1
16:1 iso G=16:1 iso ω7c	tr	tr	1.3	2.6	nd	nd
17:0 iso	1.1	tr	tr	nd	nd	tr
**Unsaturated fatty acids**						
15:1 ω6c	nd	tr	nd	nd	nd	4.0
17:1 ω8c	nd	nd	tr	tr	tr	tr
17:1 ω6c	nd	tr	tr	2.0	tr	1.4
18:1 ω9c	nd	nd	tr	nd	tr	nd
18:1 ω5c	tr	tr	nd	tr	tr	tr
**Hydroxy fatty acids (HFAs**)						
12:0 3-OH	nd	tr	tr	nd	nd	nd
15:0 2-OH	tr	tr	tr	2.6	1.3	tr
15:0 3-OH	nd	1.2	1.6	**7.5**	1.7	5.7
15:0 iso 3-OH	**8.8**	**5.9**	**6.0**	**7.4**	**10.3**	**7.0**
16:0 3-OH	tr	3.8	4.2	nd	**7.3**	2.0
16:0 iso 3-OH	tr	3.0	**5.2**	nd	**3.9**	**7.7**
17:0 2-OH (17:0 anteiso 3-OH by GC-MS)	nd	tr	tr	tr	nd	tr
17:0 3-OH	nd	nd	tr	nd	tr	nd
17:0 iso 3-OH	**17.6**	**14.4**	**11.8**	**12.4**	**14.2**	**9.1**
Summed feature*						
Sum in feature 3 (identified as the double peak of 16:1 ω7c and 15:0 iso 2-OH)	**20.9**	**21.3**	**18.3**	**25.8**	**30.8**	**22.7**
Sum in feature 9 (comment: 17:1 iso ω9c and GC-MS identified as 17:1 iso ω7c)	4.3	1.2	tr	1.6	tr	1.3
Sum in feature 4 (17:1 anteiso B/iso I identified as 17:1 iso ω5c by GC -MS)	1.3	nd	nd	nd	nd	nd
Sum in feature 2 (14:0 3-OH/16:1 iso I identified as 14:0 3-OH by GC-MS)	nd	tr	nd	nd	nd	nd

*Summed features are fatty acids that cannot be resolved reliably from another fatty acid using the chromatographic conditions chosen. The MIDI system groups these fatty acids together as one feature with a single percentage of the total.

The G+C content of the gDNA was 31.48, 30.66 and 31.98 mol% for strains 190524A05c^T^, 190524A02b^T^ and 190130A14a^T^, respectively. These values are within the reported G+C content range of the representative strains of the genus *Tenacibaculum* (38.4% for *Tenacibaculum litopenaei* and 29.6–32.2% for strains of all other species) [3].

**Fig. 2.**
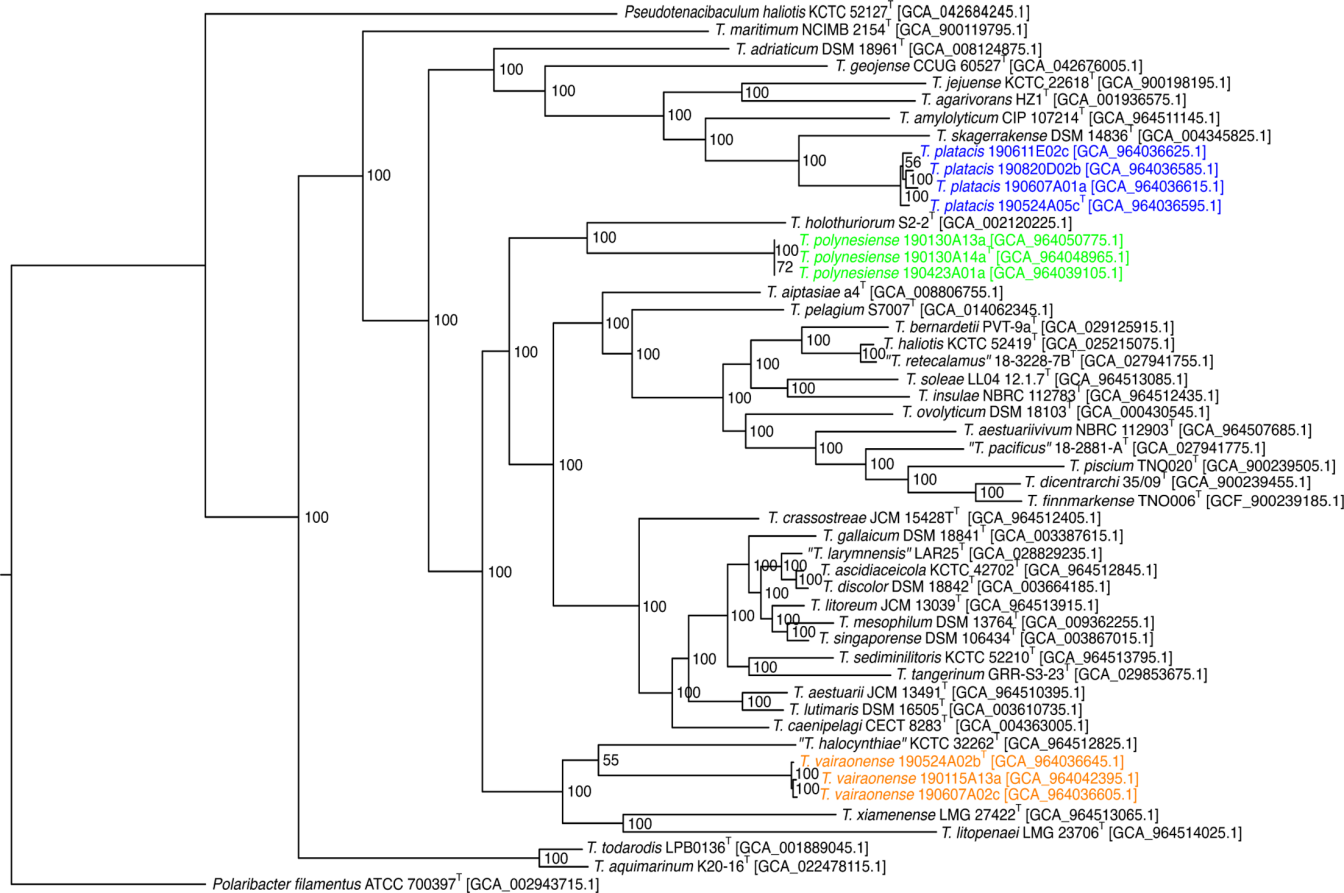
Core-genome phylogeny. The MicroScope platform was used to extract 679 single-copy core proteins from all *Tenacibaculum* type strains, and each individual alignment was concatenated using an in-house R script. Phylogenetic tree reconstruction was performed after Gblocks 0.91.1 curation using FastTree 2.1.11 and the NGPhylogeny.fr package [7]. Transfer bootstrap expectation values [24] are indicated at branch nodes.

A tentative phylogenomic core-genome tree was constructed using genomes from all species in the genus *Tenacibaculum* with the genome of *Polaribacter filamentus* ATCC 700397^T^ as an outgroup. The MicroScope platform was used to extract core proteins. A cutoff of 50% identity and 80% on the minimal coverage of the length between the aligned portions of two proteins was chosen to determine whether two coding DNA sequences (CDSs) were members of the same gene family. Six hundred seventy-nine core proteins were retrieved, and each individual alignment was concatenated using an in-house R script [17]. Core proteome tentative phylogenetic tree reconstruction was performed after Gblocks 0.91.1 curation using FastTree 2.1.11 reconstruction and the NGPhylogeny.fr package [8] with default parameters (Fig. 2). The phylogenomic tree clearly indicates that the three strains 190524A05c^T^, 190524A02b^T^ and 190130A14a^T^ are members of the genus *Tenacibaculum*. In addition, tree topology was globally congruent with the one deduced from pairwise DNA-based genome distance computation including identical neighbouring species (Fig. S2). One striking difference was the position of *T. litopenaei* within the two trees, which is likely the consequence of the biassed G+C content of its genome (38.45%) compared to those of all other *Tenacibaculum* species (29.6–32.2%). Therefore, combining the results of genomic analysis and comparisons between strains 190524A05c^T^, 190524A02b^T^ and 190130A14a^T^ with all other type strains of the genus clearly indicated that they represent three novel species of the genus *Tenacibaculum*. Genomic analyses also showed that strains 190524A05c^T^, 190611E02c, 190820D02b and 190607 A01a likely belong to the same species; that strains 190524A02b^T^, 190607 A02c and 190115 A13a belong to the same species; and that strains 190130A14a^T^, 190130 A13a and 190423 A01a form a novel species (Table 1).

## Physiology

Most morphological, physiological and biochemical tests were performed as described by Bernardet et al. [18]. The determination of growth parameters and physiological characteristics of strains 190524A05c^T^, 190524A02b^T^ and 190130A14a^T^ was performed using cells grown on marine agar (MA) or in MB at 28 °C. Colony morphology was determined using a phase-contrast optical microscope (Olympus BX40) with cells grown on MA plates for 24 h (Fig. S3). Growth was evaluated at 4, 12, 15, 18, 25, 28, 30, 34 and 37 °C and 4-day incubation time in MB. Growth at pH 5.6, 6, 6.5, 6.9, 7.5, 8, 8.4, 8.9 and 9.4 was investigated for 4 days in MB. Buffers for pH adjustment were citrate/phosphate for pH 5.0–7.0, Tris/HCl for pH 7.5–9.0 and sodium carbonate/sodium bicarbonate for pH 9.0–9.5. The salinity was tested at 0–6% NaCl (w/v) and 0.5% interval for 4 days in MB. Gliding motility was investigated on wet mounts of exponential phase MB cultures. Catalase activity was measured by observing bubble production after the application of 3% (v/v) hydrogen peroxide solution. Oxidase activity was tested using an oxidase reagent kit (bioMérieux) according to the manufacturer’s instructions. Hydrolysis of Tweens 20, 60 and 80 was determined as previously described [19]. Amylase activity was assayed on 0.2% (w/v) soluble starch MA plates. Alginate lyase activity was tested by inoculating MB solidified with 10 g l^−1^ sodium alginate according to the method of Draget *et al*. [20]. Agarase activity was tested by inoculating MA plates, while elastinase activity was assayed on MA plates supplemented with 0.75% (w/v) elastin from the bovine neck ligament as previously described [21]. The production of flexirubin-type pigments was tested by transferring bacterial material from 3-day-old cultures on a glass slide, flooding it with 20% KOH and detecting a colour change from yellow to red, violet or brown. Esterase C4, *α*-glucosidase, *β*-glucosidase and *N*-acetyl-*β*-glucosaminidase activities were performed using API ZYM galleries (bioMérieux). The phenotypic characteristics of strains 190524A05c^T^, 190524A02b^T^ and 190130A14a^T^ and related type strains were performed in parallel and are described in Table 2. In contrast with strain 190524A02b^T^, colonies of strains 190524A05c^T^ and 190130A14a^T^ showed a clear halo of hydrolysis on soluble starch MA plates, demonstrating that they possess a secreted alpha-amylase, consistent with the presence of the amylase-encoding gene *susA* in their genomes. This gene is lacking from the genome of strain 190524A02b^T^. The ability of strain 190524A05c^T^ to hydrolyse alginic acid is likely due to the presence of alginate lyase-encoding genes (*alyA1*, two copies of *alyA2*, *alyA3* and *alyA6*) similar to the ones reported in the genome of *Zobellia galactanivorans* Dsij^T^ [22]. These genes, with the exception of *alyA1* (T190524A05C_ 1530, i.e. locus_tag in the NCBI GenBank sequence available at https://www.ncbi.nlm.nih.gov/nuccore/2733071887), are encompassed in a polysaccharide utilization locus (T190524A05C_2639 to T190524A05C_2653) that also contains a predicted alginate-specific *susCD*-encoding transporter. The ability of strains 190130A14a^T^ and 190524A02b^T^ to degrade elastin is likely due to the presence of metalloprotease-encoding genes similar to elastinase FP0506 reported in the genome of *Flavobacterium psychrophilum* [21], while this gene is absent from the genome of strain 190524A05c^T^.

## Chemotaxonomy

Chemotaxonomic analyses (polar lipids, respiratory quinones and fatty acids) of strains 190524A05c^T^, 190524A02b^T^ and 190130A14a^T^, together with *Tenacibaculum aestuarii* DSM 113392^T^, *T. holothuriorum* DSM 113369 ^T^ and *T. skagerrakense* DSM 14836^T^, were carried out by the DSMZ Services, Leibniz-Institut DSMZ – Deutsche Sammlung von Mikroorganismen und Zellkulturen GmbH, Braunschweig, Germany. Fatty acid profiles of strains 190524A05c^T^, 190524A02b^T^ and 190130A14a^T^ were quite similar, characterized by the presence of significant amounts of branched, hydroxy, straight-chain and unsaturated fatty acids. The major components detected for the three strains (constituting >10% of the total fatty acids) included iso-C_15:1_ G, summed feature 3 (comprising C_16:1_ ω7c and/or iso-C_15:0_ 2-OH), iso-C_15:0_ and iso-C_17:0_ 3-OH (Table 3). The major respiratory quinone identified was menaquinone-6 (MK-6), as in all members of the family *Flavobacteriaceae*. Low levels of menaquinone-7 (MK-7; 0.5–0.7%) were also present in the three species described herein. The polar lipid profile of strain 190524A05c^T^ consisted of phosphatidylethanolamine (PE), three unidentified aminophospholipids (APLs), three unidentified aminolipids (ALs) and six unidentified lipids (Ls). Strain 190524A02b^T^ contained PE, three APLs, four ALs and four Ls, while strain 190130A14a^T^ contained PE, three APLs, four ALs), one glycolipid (GL) and four Ls (Fig. S4).

## Taxonomic conclusions

Collectively, the ANIs, dDDH, phylogenomics and phenotypic characterization of strains 190524A05c^T^, 190524A02b^T^ and 190130A14a^T^ strongly support their classification as representatives of novel species within the genera *Tenacibaculum*, for which the names *Tenacibaculum platacis* sp. nov., *Tenacibaculum vairaonense* sp. nov. and *Tenacibaculum polynesiense* sp. nov. are proposed. The three new species described in this study are distantly related to *T. maritimum*, the type species of the genus.

### Description of *Tenacibaculum platacis* sp. nov.

*Tenacibaculum platacis* (pla.ta’cis. N.L. gen. n. *platacis*, pertaining to *Platax orbicularis*, the fish species from which the strain was isolated).

Cells are Gram-negative, non-spore-forming, non-flagellated rods ~2–6 µm long and 0.3 µm in diameter motile by gliding. On MA, colonies are circular with regular edges, not adherent to the agar, glistening, yellow-orange and ~2 mm in diameter after incubation for 2 days at 28 °C. Growth in MB occurs at 18–34 °C. The pH and NaCl concentration ranges for growth are 6.5–8.0 and 1.5–5% (w/v), respectively. Oxidase positive and catalase negative. Alginate, starch, casein and Tweens (20, 60 and 80) are hydrolysed, but agar and elastin are not. Nitrate is not reduced. The major polar lipids are PE, three APLs, three ALs and six Ls. The respiratory quinones are MK-6 99.4% and MK-7 0.6%. The major fatty acids (>10% of the total fatty acids) are summed feature 3 (comprising C_16:1_ ω7c and/or iso-C_15:0_ 2-OH), iso-C_15:1_ G, iso-C_15:0_ and iso-C_17:0_ 3-OH.

The type strain, 190524A05c^T^ (= CIP 112470^T^ = DSM 118113^T^), was isolated in May 2019 from the tegument of an apparently healthy fry of *Platax orbicularis* collected in the VAIA territorial hatchery, located in Vairao, Tahiti Island, French Polynesia. The DNA G+C content of the type strain is 31.48 mol%. The GenBank accession number for the 16S rRNA gene sequence is PP620846, and the annotated complete genome sequence is deposited in the European Nucleotide Archive (ENA) under accession number GCA_964036595.1.

### Description of *Tenacibaculum vairaonense* sp. nov.

*Tenacibaculum vairaonense* (vai.ra.o.nen’se. N.L. neut. adj. *vairaonense*, pertaining to Vairao, Tahiti, the place of isolation).

Cells are Gram-negative, non-spore-forming, non-flagellated rods ~5–10 µm long and 0.5 µm in diameter motile by gliding. On MA, colonies are irregular with spreading edges, not adherent to the agar, dark yellow-orange and ~2 mm in diameter after incubation for 2 days at 28 °C. Growth in MB occurs at 18–34 °C. The pH and NaCl concentration ranges for growth are 6.5–8.0 and 1 to 4.5% (w/v), respectively. Oxidase positive and catalase negative. Casein and Tweens (20, 60 and 80) are hydrolysed, but starch, agar and alginate are not. Nitrate is not reduced. The major polar lipids are PE, three APLs, four ALs and four Ls. The respiratory quinones are MK-6 99.3% and MK-7 0.7%. The major fatty acids (>10% of the total fatty acids) are summed feature 3 (comprising C_16:1_ ω7c and/or iso-C_15:0_ 2-OH), iso-C_15:0_, iso-C_15:1_ G and iso-C_17:0_ 3-OH.

The type strain, 190524A02b^T^ (= CIP 112469^T^ = DSM 118112^T^), was isolated in May 2019 from the tegument of a young, apparently healthy fry of *Platax orbicularis* collected in the VAIA territorial hatchery, located in Vairao, Tahiti Island, French Polynesia. The DNA G+C content of the type strain is 30.66 mol%. The GenBank accession number for the 16S rRNA gene sequence is PP620847, and the annotated complete genome sequence is deposited in the ENA under accession number GCA_964036645.1.

### Description of *Tenacibaculum polynesiense* sp. nov.

*Tenacibaculum polynesiense* (po.ly.ne.si.en’se. N.L. neut. adj. *polynesiense*, pertaining to French Polynesia, where the strain was isolated).

Cells are Gram-negative, non-spore-forming, non-flagellated rods ~3–6 µm long and 0.3 µm in diameter motile by gliding. On MA, colonies are circular with regular edges, not adherent to the agar, glistering, yellow and ~2 mm in diameter after incubation for 2 days at 28 °C. Growth in MB occurs between 28 and 34 °C. The pH and NaCl concentration ranges for growth are 6.5–8.0 and 1 to 4.5% (w/v), respectively. Oxidase positive and catalase negative. Casein, starch and Tweens (20, 60 and 80) are hydrolysed, but agar and alginate are not. Nitrate is not reduced. The major polar lipids are PE, three APLs, four ALs, one GL and four Ls. The respiratory quinones are MK-6 99.5% and MK-7 0.5%. The major fatty acids (>10% of the total fatty acids) are iso-C_15:1_ G, summed feature 3 (comprising C_16:1_ ω7c and/or iso-C_15:0_ 2-OH), iso-C_15:0_ and iso-C_17:0_ 3-OH.

The type strain, 190130A14a^T^ (= CIP 112468^T^ = DSM 118111^T^), was isolated in May 2019 from the tegument of a young, apparently healthy fry of *Platax orbicularis* collected in the VAIA territorial hatchery, located in Vairao, on the island of Tahiti in French Polynesia. The DNA G+C content of the type strain is 31.98 mol%. The GenBank accession number for the 16S rRNA gene sequence is PP620848, and the annotated complete genome sequence is deposited in the EMBL under accession number GCA_964048965.1.

## Supplementary material

10.1099/ijsem.0.006605 Uncited Fig. S1.

## Abbreviations

ALs: unidentified aminolipids
ANI: average nucleotide identity
APLs: unidentified aminophospholipids
dDDH: digital DNA–DNA hybridization
ENA: European Nucleotide Archive
FSMA: *Flavobacteriaceae*-selective marine agar
gDNA: genomic DNA
GL: glycolipid
Ls: unidentified lipids
MA: marine agar
MB: marine broth
MK-6: menaquinone-6
MK-7: menaquinone-7
PE: phosphatidylethanolamine
