# Supplementary material for: Tenacibaculum platacis sp. nov., Tenacibaculum vairaonense sp. nov. and Tenacibaculum polynesiense sp. nov. isolated from batfish (Platax orbicularis) in Tahiti Island, French Polynesia
Source: Int J Syst Evol Microbiol. 2025 Jan 6;75(1):006605. doi: 10.1099/ijsem.0.006605 (PMC12281758; doi:10.1099/ijsem.0.006605)
Supplement: Uncited Fig. S1. [file ijsem-75-06605-s001.pdf]

SUPPLEMENTARY INFORMATION

***Tenacibaculum platacis* sp. nov., *Tenacibaculum vairaonense* sp. nov. and *Tenacibaculum polynesiense* sp. nov. isolated from batfish (*Platax orbicularis)* in Tahiti Island, French Polynesia.**

Pierre Lopez[1-2], Benjamin Fradet[2], Lucie Coffion[3], Jean-François Bernardet[2], Denis Saulnier[1] & Eric Duchaud[2]

[1] Ifremer, IRD, Institut Louis-Malardé, Université Polynésie française, EIO, F-98719 Taravao, Tahiti, Polynésie française, France.
[2] Université Paris-Saclay, INRAE, UVSQ, VIM, Jouy-en-Josas, France.
[3] LABGeM, Génomique Métabolique, CEA, Genoscope, Institut François Jacob, Université d'Évry, Université Paris-Saclay, CNRS, Evry, France.

Supplementary figures:

Supplementary figure 1: Maximum-Likelihood 16S rRNA-based phylogeny. 2

Supplementary figure 2: ANI-based tree. 3

Supplementary figure 3: Colony morphology of *Tenacibaculum* strains. 4

Supplementary figure 4: Chromatograms of the total polar lipids. 5

Supplementary tables:

Supplementary table 1: Genome characteristics. 6

Supplementary table 2: OrthoANI values. 7

Supplementary table 3: dDDH values. 8

Supplementary Figure 1: Maximum-Likelihood 16S rRNA-based phylogeny. Phylogenetic tree inferred by PhyML using the 16S rRNA of *Tenacibaculum* species. The type strain of *Pseudotenacibaculum haliotis* has been included and the type strain of *Polaribacter filamentus* was used as an outgroup. Scale bar indicates estimated sequence divergence and bootstrap values are indicated at branch nodes. The 16S rRNA sequence of strains *"T. larymnensis"* LAR25^T, *"T. pacificus"* 18-2881-A^T and *"T. retecalamus"* 18-3228-7B^T have been retrieved from complete genomes accession number GCF_028829235.1, NZ_CP115917 and NZ_CP115916, respectively

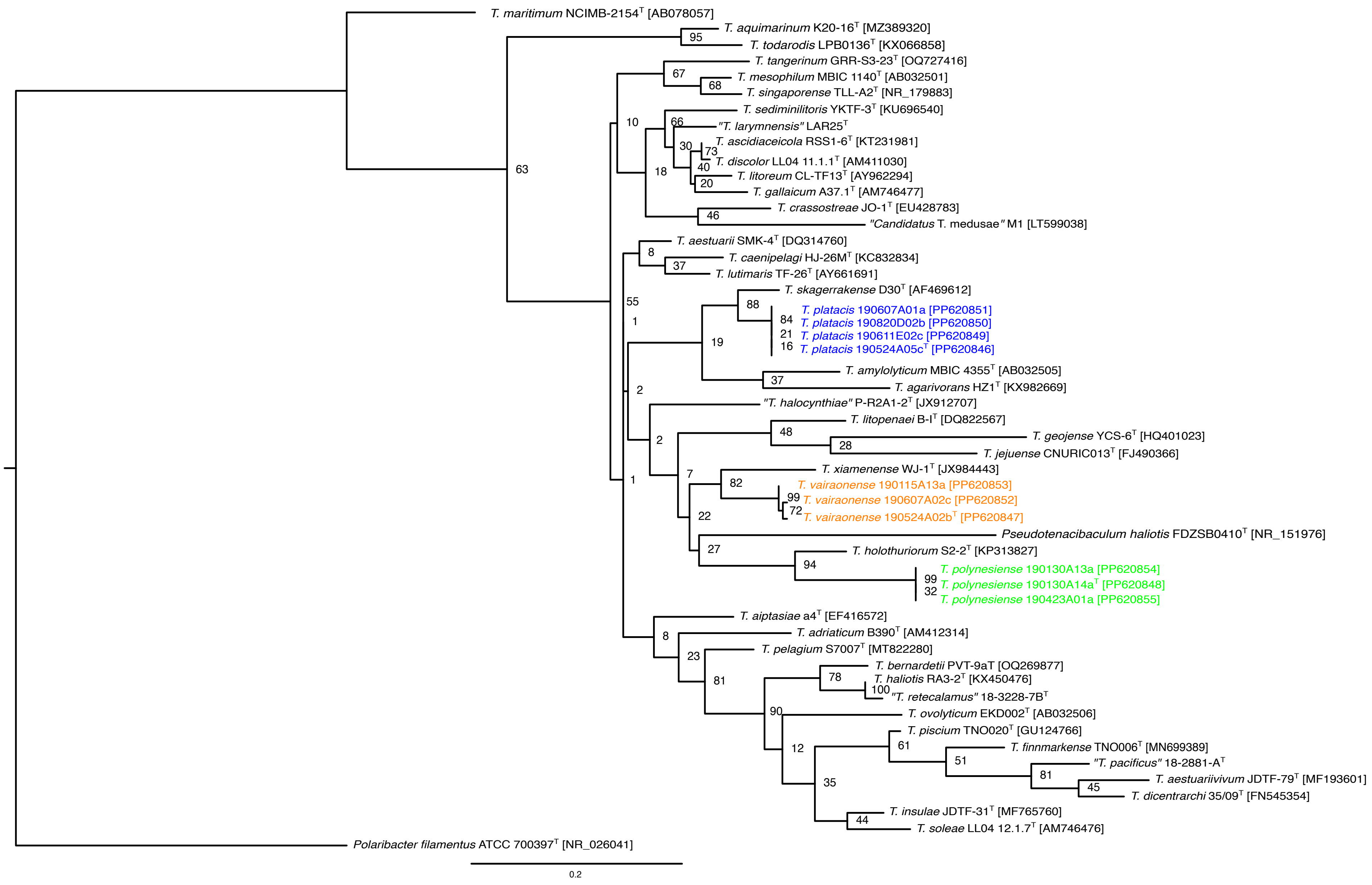

Supplementary figure 2: ANI-based phylogenetic tree.Genomic similarity was estimated using the Mash software that computes a distance between two genomes. A tree was constructed from all thepairwise distances of the genome set using the neighbour-joining Java script package V 1.0.4 (https://github.com/biosustain/neighbor-joining). MASHdistances are indicated on tree branches.

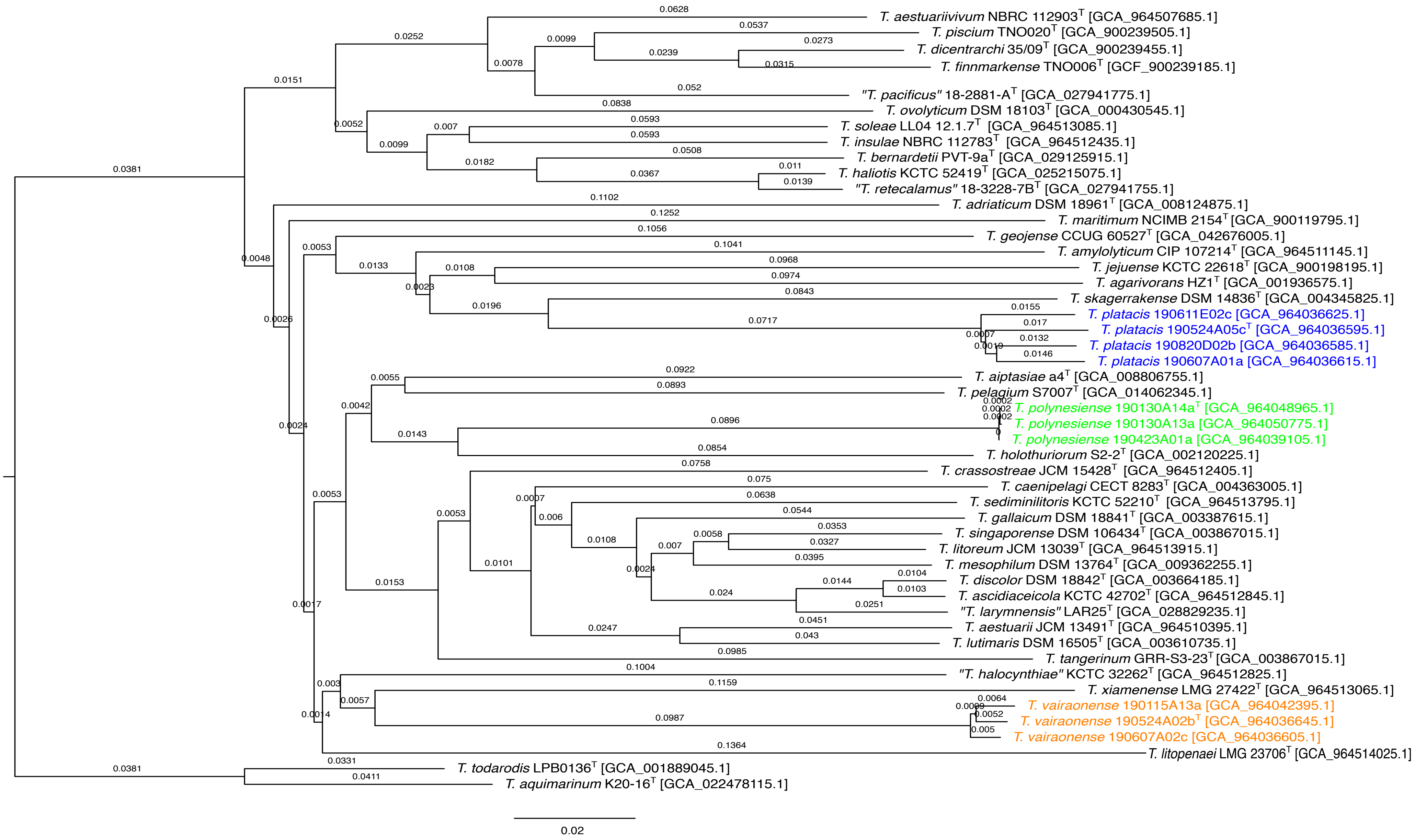

Supplementary figure 3. Colony morphology of *Tenacibaculum* strains grown on MA plates for 24h at 28°C and observed by phase-contrast microscopy. (A) *T. platacis* sp. nov. 190524A05c^{T}, (B) *T. vairaonense* sp. nov. 190524A02b^{T}, (C) *T. polynesiense* sp. nov. 190130A14a^{T}.

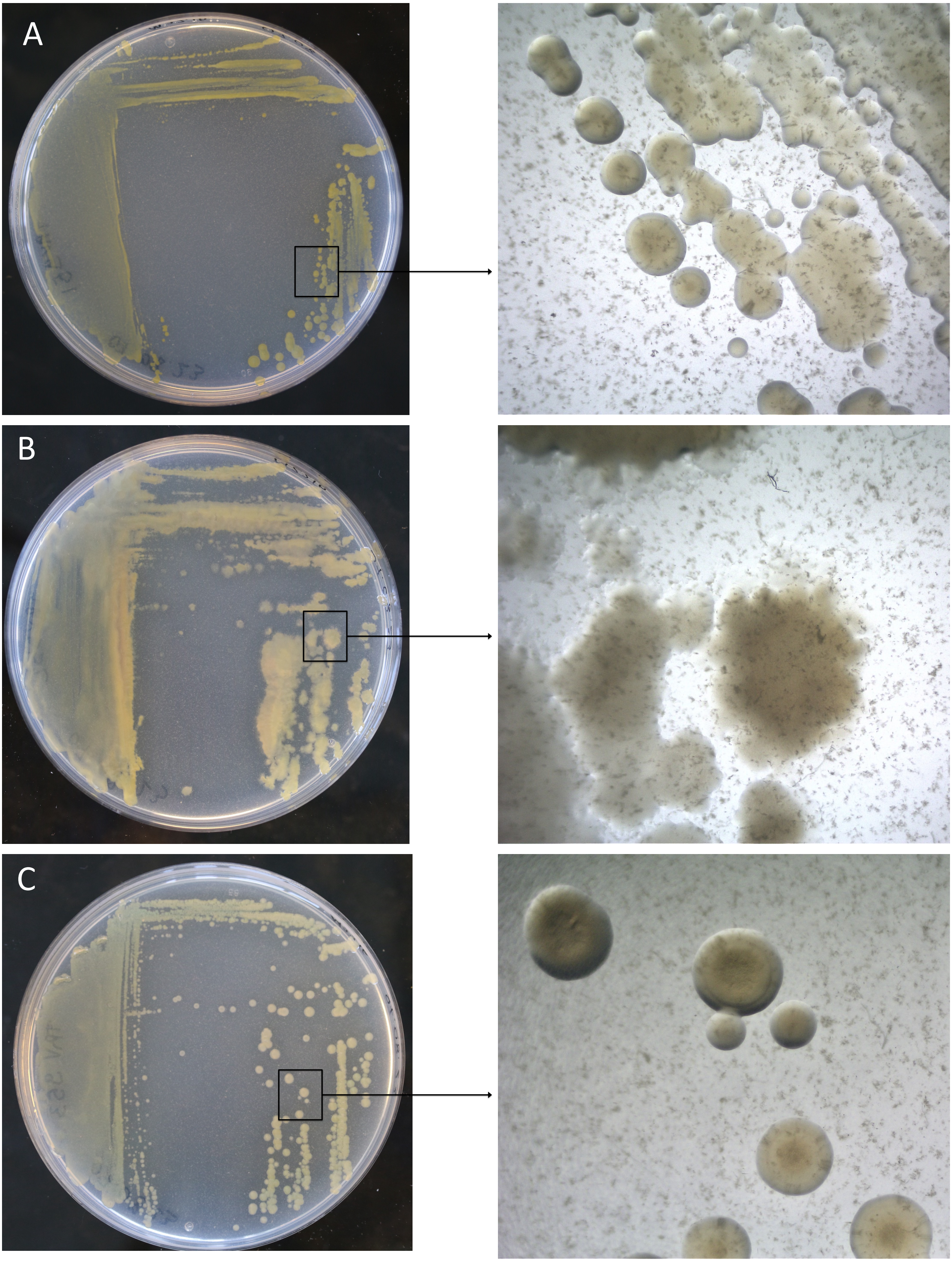

Supplementary figure 4: Two-dimensional thin-layer chromatograms of the total polar lipids of strains 190130A14a^{T} (A), 190524A02b^{T} (B) and 190524A05c^{T} (C). AL, unidentified aminolipids; APL, unidentified aminophospholipids; GL, unidentified glycolipid; PE, phosphatidylethanolamine; PL, unidentified phospholipid; L, unidentified polar lipids.

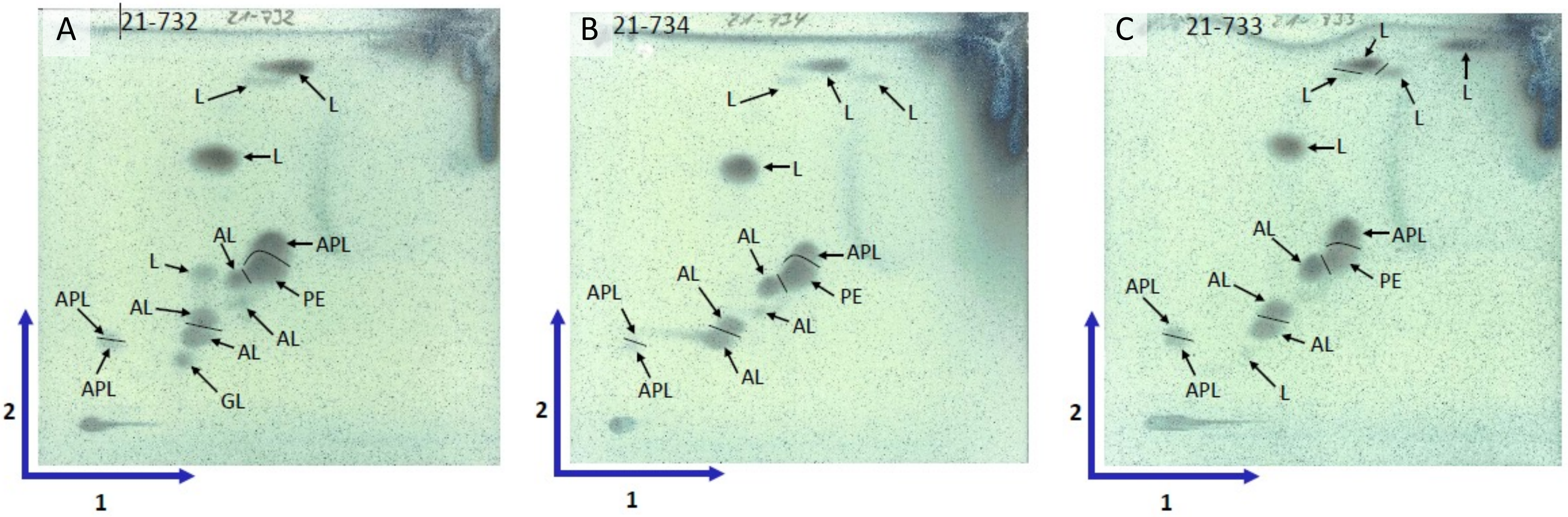

Supplementary Table 1 : Genome characteristics

Species	Strain	Assembly size (bp)	G+C content (mol%)	Number of contigs (>2kb)	N50 (bp)	Completeness (%)	Contamination (%)	Average long read (Nanopore) coverage	Average short read (Illumina) coverage	Number of coding sequences (CDS)	Number of tRNA	Number of rRNA operons	ENA accession number
Tenacibaculum platacis	**190524A05c[T]**	4,130,482	31.48	1	4,130,482	99.6644	0.159795	149	750	3,615	54	3	GCA_964036595.1
	**190611E02c**	4,042,826	31.42	14	874,873	99.6644	0.243688		459	3,707	nd	nd	GCA_964036625.1
	**190820D02b**	4,011,514	31.46	19	455,254	99.6644	0.419463		542	3,652	nd	nd	GCA_964036585.1
	**190607A01a**	4,016,071	31.46	11	938,204	99.6644	0		427	3,622	nd	nd	GCA_964036615.1
Tenacibaculum vairaonense	**190524A02b[T]**	5,036,910	30.66	1	5,036,910	99.6644	1.566	97	346	4,085	68	6	GCA_964036645.1
	**190115A13a**	4,802,973	30.40	46	246,949	99.6644	1.00671		412	4,121	nd	nd	GCA_964042395.1
	**190607A02c**	4,767,073	30.40	41	258,17	99.6644	1.36465		374	4,096	nd	nd	GCA_964036605.1
Tenacibaculum polynesiense	**190130A14a[T]**	3,936,153	31.98	1	3,936,153	99.6644	1.59396	166	2,205	3,578	65	5	GCA_964048965.1
	**190130A13a**	3,843,691	31.73	18	569,163	99.6644	1.59396		460	3,700	nd	nd	GCA_964050775.1
	**190423A01a**	3,843,974	31.73	18	568,14	99.6644	1.59396		420	3,706	nd	nd	GCA_964039105.1

Supplementary Table 2: OrthoANI values between all the *Tenacibaculum* type strains described to date and the strains isolated during this study. Quoted names have not been validly published. OrthoANI values were obtained using the OAT software version 0,93,1. The proposed cut-off for species demarcation is 95~96% for OrthoANI. The detailed algorithm is given in Lee et al. 2016.

	Polaribacter filamentus ATCC 700397^{T}	*Pseudotenacibaculum haliotis* FDZSB041^{T}	"*T. halocynthiae*" KCTC 32262^{T}	"*T. larymnensis*" LAR25^{T}	"*T. pacificus*" 18-2881-A^{T}	"*T. retecalamus*" 18-3228-7B^{T}	*T. adriaticum* B390^{T}	*T. aestuarii* SMK-4^{T}	*T. aestuariivivum* NBRC 112903^{T}	*T. agarivorans* HZ1^{T}	*T. aiptasiae* a4^{T}	*T. amylolyticum* CIP 107214^{T}	*T. aquimarinum* K20-16^{T}	*T. ascidiaceicola* KCTC 42702^{T}	*T. bernardetii* PVT-9a^{T}	*T. caenipelagi* KCTC 32323^{T}	*T. crassostreae* JO1^{T}	*T. dicentrarchi* 35/09^{T}	*T. discolor* DSM 18842^{T}	*T. finnmarkense* TNO006^{T}	*T. gallaicum* A37.1^{T}	*T. geojense* YCS6^{T}	*T. haliotis* NBRC 112382^{T}	*T. holothuriorum* LMG 27758^{T}	*T. insulae* NBRC 112783^{T}	*T. jejuense* KCTC 22618^{T}	*T. litopenaei* LMG 23706^{T}	*T. litoreum* CL-TF13^{T}	*T. lutimaris* TF-26^{T}	*T. maritimum* NCIMB 2154^{T}	*T. mesophilum* CIP 107215^{T}	*T. ovolyticum* EKD002^{T}	*T. platacis* 190524A05c^{T}	*T. platacis* 190607A01a	*T. platacis* 190611E02c	*T. platacis* 190820D02b	*T. pelagium* S7007^{T}	*T. piscium* TNO020^{T}	*T. polynesiense* 190130A14a^{T}	*T. polynesiense* 190130A13a	*T. polynesiense* 190423A01a	*T. sediminilitoris* KCTC 52210^{T}	*T. singaporense* DSM 106434^{T}	*T. skagerrakense* DSM 14836^{T}	*T. soleae* LL04 12.1.7^{T}	*T. tangerinum* GRR-S3-23^{T}	*T. todarodis* LPB0136^{T}	*T. vairaoense* 190524A02b^{T}	*T. vairaoense* 190115A13a	*T. vairaoense* 190607A02c	*T. xiamenense* LMG 27422^{T}
Polaribacter filamentus ATCC 700397^{T}	100
Pseudotenacibaculum haliotis FDZSB041^{T}	72	100
"*T. halocynthiae*" KCTC 32262^{T}	74	72	100
"*T. larymnensis*" LAR25^{T}	74	73	77	100
"*T. pacificus*" 18-2881-A^{T}	75	72	78	78	100
"*T. retecalamus*" 18-3228-7B^{T}	75	72	77	78	83	100
T. adriaticum B390^{T}	74	73	76	77	76	77	100
T. aestuarii SMK-4^{T}	74	73	77	85	78	78	77	100
T. aestuariivivum NBRC 112903^{T}	75	72	78	78	86	82	76	78	100
T. agarivorans HZ1^{T}	73	72	74	75	75	75	75	75	75	100
T. aiptasiae a4^{T}	74	73	77	79	79	79	76	79	79	75	100
T. amylolyticum CIP 107214^{T}	73	72	74	75	75	75	76	75	75	77	75	100
T. aquimarinum K20-16^{T}	77	72	75	75	77	77	76	76	77	74	76	74	100
T. ascidiaceicola KCTC 42702^{T}	74	73	77	95	78	78	77	85	78	75	79	75	75	100
T. bernardetii PVT-9a^{T}	75	72	77	78	82	88	76	78	82	74	79	75	77	78	100
T. caenipelagi KCTC 32323^{T}	74	73	77	84	78	78	77	85	78	75	79	75	76	84	78	100
T. crassostreae JO1^{T}	74	73	77	82	78	78	77	83	78	75	79	75	76	82	78	82	100
T. dicentrarchi 35/09^{T}	75	72	77	77	87	82	76	78	84	74	79	74	76	77	81	77	78	100
T. discolor DSM 18842^{T}	74	73	77	95	78	78	76	85	78	75	79	75	76	98	78	84	82	77	100
T. finnmarkense TNO006^{T}	74	72	76	77	87	81	76	77	83	74	78	74	76	77	81	77	77	93	77	100
T. gallaicum A37.1^{T}	74	73	77	89	78	78	76	84	78	75	78	75	75	89	78	84	82	77	89	77	100
T. geojense YCS6^{T}	73	72	75	75	75	75	76	75	75	75	75	75	74	75	75	75	76	75	75	75	75	100
T. haliotis NBRC 112382^{T}	75	72	77	78	83	97	76	78	83	75	80	75	77	78	88	78	78	82	78	81	78	75	100
T. holothuriorum LMG 27758^{T}	74	73	76	78	77	77	76	78	77	75	78	75	76	78	77	77	78	76	78	76	77	75	77	100
T. insulae NBRC 112783^{T}	75	72	77	79	82	85	77	78	82	75	80	75	77	79	83	78	79	81	79	81	78	75	85	77	100
T. jejuense KCTC 22618^{T}	73	72	74	75	75	75	75	75	75	79	75	77	74	75	74	75	75	74	75	74	75	75	75	75	75	100
T. litopenaei LMG 23706^{T}	70	70	72	73	72	72	72	73	72	71	72	71	71	72	72	73	73	72	73	72	72	71	72	72	72	71	100
T. litoreum CL-TF13^{T}	74	73	77	90	78	78	77	86	78	75	79	75	76	90	78	85	82	77	90	77	88	76	78	78	78	75	72	100
T. lutimaris TF-26^{T}	74	73	77	87	78	78	77	90	78	75	79	75	76	88	78	84	83	77	88	77	85	76	78	78	79	75	73	86	100
T. maritimum NCIMB 2154^{T}	73	72	75	75	75	75	75	75	75	73	75	73	75	75	75	75	75	75	75	75	75	74	75	75	75	73	71	75	75	100
T. mesophilum CIP 107215^{T}	74	73	77	89	78	78	76	85	78	75	78	75	76	89	78	84	82	77	89	77	89	75	78	77	78	75	72	90	85	75	100
T. ovolyticum EKD002^{T}	75	72	77	78	82	83	76	78	82	74	79	74	76	78	82	77	78	81	78	81	77	75	83	77	82	74	71	78	78	75	77	100
T. platacis 190524A05c^{T}	73	72	74	75	75	74	75	75	74	77	75	77	74	75	74	75	75	74	75	74	75	75	74	75	75	77	71	75	75	73	75	74	100
T. platacis 190607A01a	73	72	74	75	75	75	75	75	74	77	75	77	74	75	74	75	75	74	75	74	75	75	75	75	75	77	71	75	75	73	75	74	96	100
T. platacis 190611E02c	73	72	74	75	75	74	75	75	75	77	75	78	74	75	74	75	75	74	75	74	75	75	74	75	75	77	71	75	75	73	75	74	96	97	100
T. platacis 190820D02b	73	72	74	75	75	75	75	75	74	77	75	77	74	75	74	75	75	74	75	74	75	75	74	75	75	77	71	75	75	73	75	74	96	97	97	100
T. pelagium S7007^{T}	75	73	78	79	80	81	77	79	80	75	81	76	77	79	80	79	80	79	79	79	79	76	81	78	81	75	72	79	79	76	79	80	75	75	75	75	100
T. piscium TNO020^{T}	75	72	76	77	85	81	76	77	83	74	78	74	76	77	81	77	77	86	77	86	77	74	81	76	80	74	72	77	77	75	77	80	74	74	74	74	79	100
T. polynesiense 190130A14a^{T}	73	72	76	77	76	76	75	77	76	75	77	75	75	77	76	77	79	76	77	76	77	75	76	79	77	75	72	77	77	74	77	76	74	74	75	75	78	76	100
T. polynesiense 190130A13a	73	72	76	77	76	76	75	77	76	74	77	75	75	77	76	77	78	76	77	76	77	75	76	79	77	75	72	77	77	74	77	76	74	74	74	75	78	76	100	100
T. polynesiense 190423A01a	73	72	76	77	77	76	75	77	76	74	77	75	75	78	76	77	79	76	77	76	77	75	76	79	77	75	72	77	77	74	77	76	75	74	74	75	78	76	100	100	100
T. sediminilitoris KCTC 52210^{T}	74	72	77	86	78	78	76	84	78	74	78	75	75	87	78	84	82	77	87	77	86	75	78	77	79	75	73	88	84	75	86	78	75	75	74	75	79	77	77	77	77	100
T. singaporense DSM 106434^{T}	74	73	77	91	78	78	77	86	78	75	79	75	76	90	78	85	82	77	90	77	88	75	78	78	79	75	73	93	86	75	91	78	75	75	75	75	79	77	78	78	77	87	100
T. skagerrakense DSM 14836^{T}	73	72	74	75	75	75	76	75	75	77	75	77	74	75	74	75	75	74	75	74	75	75	75	75	75	77	71	75	75	73	75	74	81	81	81	81	75	74	75	75	75	75	75	100
T. soleae LL04 12.1.7^{T}	75	72	77	78	82	85	77	78	82	74	79	75	77	78	83	78	78	81	78	80	78	75	86	77	85	75	72	78	78	75	78	83	74	74	74	74	80	80	76	76	76	78	78	75	100
T. tangerinum GRR-S3-23^{T}	73	72	76	83	77	77	76	82	77	74	77	74	75	83	77	81	80	77	83	76	83	75	77	76	77	74	73	84	82	74	83	77	74	74	74	74	78	76	76	76	76	83	83	74	77	100
T. todarodis LPB0136^{T}	73	73	75	76	77	77	76	75	77	74	76	74	91	75	77	76	76	76	76	76	75	75	77	76	77	74	71	75	76	75	75	77	74	74	74	74	77	76	75	75	75	75	75	74	77	75	100
T. vairaoense 190524A02b^{T}	73	72	77	76	76	76	75	77	76	74	76	74	75	76	76	76	77	76	76	75	76	75	76	76	76	74	72	76	77	74	76	75	74	74	74	74	77	75	76	76	76	76	76	74	76	76	75	100
T. vairaoense 190115A13a	73	72	77	76	76	76	75	77	76	74	76	75	75	77	76	76	77	76	77	75	76	75	76	76	76	74	72	76	77	74	77	75	74	74	74	74	77	76	76	76	76	76	76	74	76	76	75	99	100
T. vairaoense 190607A02c	73	72	77	76	76	76	75	77	76	74	76	75	75	76	76	77	77	76	76	75	76	75	76	76	76	74	72	76	77	74	76	75	74	74	74	74	77	75	76	76	76	76	76	74	76	76	75	99	99	100
T. xiamenense LMG 27422^{T}	72	71	75	75	75	74	74	75	75	73	75	73	73	75	76	75	75	74	75	74	75	74	74	75	75	73	72	75	75	73	75	74	73	73	73	73	75	74	75	75	75	75	75	73	75	76	73	76	76	76	100

Color scale
98
95
90
85
80

x

Supplementary Table 3: dDDH values between *T. platacis* 190524A05c[T]; *T. polynesiense* 190130A14a[T]; *T. vairaonense* 190524A02b[T] and the *Tenacibaculum* type strains using Genome-to-Genome Distance Calculator 3.0 and Formula: 2 identities / HSP length. Quoted names have not been validly published.

	T. platacis 190524A05c	*T. polynesiense* 190130A14a	*T. vairaonense* 190524A02b
***Polaribacter filamentus* ATCC 700397[T]**	18.90%	19.50%	20.20%
***Pseudotenacibaculum haliotis* FDZSB041[T]**	18.80%	18.70%	20.30%
**"*T. halocynthiae*" KCTC 32262[T]**	19.00%	20.00%	21.40%
**"*T. larymnensis*" LAR25[T]**	19.70%	20.70%	20.80%
**"*T. pacificus*" 18-2881-A[T]**	19.80%	20.50%	21.00%
**"*T. retecalamus*" 18-3228-7B[T]**	19.70%	20.20%	20.70%
***T. adriaticum* B390[T]**	19.20%	20.60%	20.40%
***T. aestuarii* SMK-4[T]**	20.00%	20.70%	21.00%
***T. aestuariivivum* NBRC 112903[T]**	20.00%	20.70%	21.00%
***T. agarivorans* HZ1[T]**	21.40%	19.80%	20.20%
***T. aiptasiae* a4[T]**	19.70%	20.70%	20.70%
***T. amylolyticum* CIP 107214[T]**	21.00%	20.30%	20.10%
***T. aquimarinum* K20-16[T]**	18.30%	19.40%	19.80%
***T. ascidiaceicola* KCTC 42702[T]**	19.90%	21.20%	21.10%
***T. bernardetii* PVT-9a[T]**	18.60%	19.80%	20.40%
***T. caenipelagi* KCTC 32323[T]**	19.50%	20.90%	20.50%
***T. crassostreae* JO1[T]**	19.70%	22.00%	21.20%
***T. dicentrarchi* 35/09[T]**	20.70%	20.30%	21.20%
***T. discolor* DSM 18842[T]**	19.90%	21.20%	21.10%
***T. finnmarkense* TNO006[T]**	19.20%	19.70%	20.40%
***T. gallaicum* A37.1[T]**	19.60%	20.80%	20.90%
***T. geojense* YCS6[T]**	19.70%	19.80%	20.30%
***T. haliotis* NBRC 112382[T]**	19.70%	20.40%	20.70%
***T. holothuriorum* LMG 27758[T]**	19.60%	22.60%	20.40%
***T. insulae* NBRC 112783[T]**	19.10%	20.20%	21.00%
***T. jejuense* KCTC 22618[T]**	20.70%	19.70%	20.70%
***T. litopenaei* LMG 23706[T]**	18.80%	18.60%	18.50%
***T. litoreum* CL-TF13[T]**	19.70%	20.70%	21.10%
***T. lutimaris* TF-26[T]**	19.90%	20.70%	21.10%
***T. maritimum* NCIMB 2154[T]**	19.00%	19.70%	19.80%
***T. mesophilum* CIP 107215[T]**	20.00%	21.10%	21.10%
***T. ovolyticum* EKD002[T]**	19.30%	20.20%	21.10%
***T. platacis* 190524A05c[T]**		19.90%	20.10%
***T. platacis* 190607A01a**	69.20%	19.90%	20.10%
***T. platacis* 190611E02c**	68.50%	19.70%	20.30%
***T. platacis* 190820D02b**	69.20%	19.70%	20.20%
***T. pelagium* S7007[T]**	19.70%	20.90%	20.80%
***T. piscium* TNO020[T]**	19.80%	19.70%	20.60%
***T. polynesiense* 190130A14a[T]**	19.70%		21.90%
***T. polynesiense* 190130A13a**	19.70%	99.60%	21.70%
***T. polynesiense* 190423A01a**	19.70%	99.50%	21.70%
***T. sediminilitoris* KCTC 52210[T]**	19.10%	20.60%	20.70%
***T. singaporense* DSM 106434[T]**	19.80%	21.20%	21.50%
***T. skagerrakense* DSM 14836[T]**	24.10%	19.70%	20.20%
***T. soleae* LL04 12.1.7[T]**	19.30%	20.10%	20.70%
***T. tangerinum* GRR-S3-23[T]**	19.60%	20.50%	20.60%
***T. todarodis* LPB0136[T]**	18.70%	19.50%	20.50%
***T. vairaonense* 190524A02b[T]**	20.10%	21.90%	
***T. vairaonense* 190115A13a**	19.60%	20.80%	89.00%
***T. vairaonense* 190607A02c**	19.90%	21.00%	88.20%
***T. xiamenense* LMG 27422[T]**	19.60%	20.00%	19.80%
